# Impact of conflict on infant immunisation coverage in Afghanistan: a countrywide study 2000–2003

**DOI:** 10.1186/1476-072X-6-23

**Published:** 2007-06-07

**Authors:** Taufiq Mashal, Keiko Nakamura, Masashi Kizuki, Kaoruko Seino, Takehito Takano

**Affiliations:** 1 Health Promotion Section, Division of Public Health, Graduate School of Tokyo Medical and Dental University, Tokyo, Japan; 2 International Health Section, Division of Public Health, Graduate School of Tokyo Medical and Dental University, Tokyo, Japan

## Abstract

**Background:**

Infant immunisation is an effective public health intervention to reduce the morbidity and mortality of vaccine preventable diseases. However, some developing countries fail to achieve desirable vaccination coverage; Afghanistan is one such country. The present study was performed to evaluate the progress and variation in infant immunisation coverage by district and region in Afghanistan and to assess the impact of conflict and resource availability on immunisation coverage.

**Results:**

This study analysed reports of infant immunisation from 331 districts across 7 regions of Afghanistan between 2000 and 2003. Geographic information system (GIS) analysis was used to visualise the distribution of immunisation coverage in districts and to identify geographic inequalities in the process of improvement of infant immunisation coverage. The number of districts reporting immunisation coverage increased substantially during the four years of the study. Progress in Bacillus Calmette-Guerin (BCG) immunisation coverage was observed in all 7 regions, although satisfactory coverage of 80% remained unequally distributed. Progress in the third dose of Diphtheria-Pertussis-Tetanus (DPT3) immunisation differed among regions, in addition to the unequal distribution of immunisation coverage in 2000. The results of multivariate logistic regression analysis indicated a significant negative association between lack of security in the region and achievement of 80% coverage of immunisation regardless of available resources for immunisation, while resource availability showed no relation to immunisation coverage.

**Conclusion:**

Although progress was observed in all 7 regions, geographic inequalities in these improvements remain a cause for concern. The results of the present study indicated that security within a country is an important factor for affecting the delivery of immunisation services.

## Background

More than 95% of the 14 million deaths of children under 5 years old around the world occur in developing countries. Moreover, at least 70% of these deaths are due to diseases that can be prevented by vaccination [1,2]. Of 165 countries where immunisation coverage data are available, 20% have failed to achieve 80% coverage of Diphtheria-Pertussis-Tetanus (DPT) immunisation for infants. Moreover, 10% of these countries have failed to achieve even 50% coverage [3]. Afghanistan is one such country, which has not achieved reasonable coverage of infant immunisation.

Provision of immunisation services to all children is regarded as a priority of basic health services. However, poverty and lack of basic health services prevent appropriate immunization of children against preventable illnesses [4,5]. A country's public health system, including immunisation programs, can be easily devastated by armed conflicts [6]. In 2003 alone, there were 29 armed conflicts active in 22 countries around the world, the majority of which were intrastate conflicts [7]. Most such conflicts occur in developing countries, and children are among the worst-affected victims [8-10]. Countries continue struggling to provide immunisation services despite the lack of security, but there have been few studies, in general, regarding the influence of insecurity within a country on child immunisation coverage [11].

The child immunisation program in Afghanistan has a long history under the name of the "Mass Immunisation Program". The Ministry of Public Health re-organised the program in 1978 as the "Expanded Program of Immunisation (EPI) Program", and focused on expansion of immunisation coverage along with the introduction of new vaccinations [12]. Efforts to restructure EPI began in 1994 when the first immunisation campaign was initiated, and the regional system of EPI management was implemented in 1995 [13]. A national organisational structure for the management and implementation of immunisation services has been developed and maintained by the Ministry of Public Health with the collaboration of international organisations [14]. Although a national scheme was developed to cover all districts within the country, implementation to cover various areas in the country has started only recently.

People of Afghanistan had enjoyed a period of peaceful life by 1978, when armed conflict prevail after Soviet troop's presence in the country and continue up to 1992. Thereafter a very unstable period with factions fighting each other was followed by a relatively stable period during which the Taliban ruled most of the country, a period that was ended by a military intervention of the international community at the end of 2001. Since then, despite its official classification as 'post-conflict', the security situation remains fragile in many parts of Afghanistan [15,16]. Child health in Afghanistan has become a problem of particular concern due to the protracted period of conflict within the country [17].

The effective and efficient provision of immunisation to all those in need of such services is now a major priority in Afghanistan. However, both direct and indirect influences of conflict over time in Afghanistan have adversely affected immunisation programs. Despite efforts at various levels, there is particular concern regarding the negative impact of continuing insecurity in the country on immunisation services.

The present study was performed to evaluate the progress and variation in infant immunisation coverage by district and region in Afghanistan and to assess the impact of conflict and resource availability on immunisation coverage. Analysis of infant immunisation coverage and conflicts by district and region in Afghanistan during the period of vast changes in security will provide critical evidence regarding immunisation under conditions of conflict. The study will also provide a practical example of the use of countrywide health data for the assessment of immunisation services in post-conflict countries with limited resources.

## Methods

### Settings

Afghanistan is one of the less well-developed countries in South Asia. The central and eastern parts of the country, covering about three-quarters of the land area, are dominated by mountains. In 2002, the country had a population of 22 million people with diverse and traditional cultural backgrounds [18], and more than 80% of the population lived in rural areas. With the collaboration of the government of Afghanistan, the World Health Organization country office for Afghanistan defined 331 districts and 7 regions, which were then updated by the Afghanistan Information Management Service (AIMS). The present study was carried out using this classification of districts as units of analysis.

### Immunisation services

By 2000, the following vaccinations were scheduled for children before the age of 12 months: one dose of Bacillus Calmette-Guerin (BCG) at birth or on first contact by a health worker, three doses of DPT (DPT1, DPT2 and DPT3) beginning from 6 weeks of age and at 4-week intervals, three doses of oral polio vaccine (OPV1, OPV2 and POV3) according to the same schedule as DPT and one dose of measles vaccine at the age of 9 months [14]. In 2003, the following organisations were responsible for planning, secure supply and logistics, monitoring activities by collecting data regarding implementation, supervision to maintain quality of service and financial management: one National EPI Office at Ministry of Public Health, 7 regional EPI Management Teams, and 32 provincial EPI Management Teams in collaboration with the World Health Organization (WHO) and United Nations Children's Fund (UNICEF) [14]. A total of 722 EPI centres in 331 districts acted as direct providers of immunisation services. According to the national guidelines, the administration of all scheduled vaccines is carried out by these centres, which includes standard service of fixed (within-centre, two days per week) and outreach (somewhere in the community, four days per week) activities [14].

### Data sources

In the present study, we used databases of the reports of immunisation coverage and resources by 331 districts of Afghanistan in 2000 and in 2003 shared by UNICEF and WHO country offices in Afghanistan. These two offices were working in collaboration with the Ministry of Public Health, Afghanistan, to implement the EPI program and had been providing financial and technical support. Databases were developed according to the immunisation hierarchical reporting system, and included district, provincial, regional and national institutions involved in implementation of immunisations. Well-trained vaccinators assigned to individual EPI centres recorded immunisation practices on national standard reporting forms. These completed forms were then submitted to the provincial EPI supervisor, and then reported to regional and national coordinators. A country-wide database was developed based on these reports. The vaccinators were all responsible for immunisations in the assigned areas, and their direct reporting was regarded as reflecting the actual services reaching children in individual communities.

### Study variables

National infant immunisation coverage rates for the four vaccines (BCG, DPT3, POV3 and measles) were calculated for the years 2000 and 2003, according to the following formula:

Immunisation coverage rate for ["X" scheduled vaccine]:

Reported number of children under 12 months of age who received "X" scheduled vaccineEstimated number of children under 12 months of age×100
 MathType@MTEF@5@5@+=feaafiart1ev1aaatCvAUfKttLearuWrP9MDH5MBPbIqV92AaeXatLxBI9gBaebbnrfifHhDYfgasaacH8akY=wiFfYdH8Gipec8Eeeu0xXdbba9frFj0=OqFfea0dXdd9vqai=hGuQ8kuc9pgc9s8qqaq=dirpe0xb9q8qiLsFr0=vr0=vr0dc8meaabaqaciaacaGaaeqabaqabeGadaaakeaadaWcaaqaaiabbkfasjabbwgaLjabbchaWjabb+gaVjabbkhaYjabbsha0jabbwgaLjabbsgaKjabbccaGiabb6gaUjabbwha1jabb2gaTjabbkgaIjabbwgaLjabbkhaYjabbccaGiabb+gaVjabbAgaMjabbccaGiabbogaJjabbIgaOjabbMgaPjabbYgaSjabbsgaKjabbkhaYjabbwgaLjabb6gaUjabbccaGiabbwha1jabb6gaUjabbsgaKjabbwgaLjabbkhaYjabbccaGiabbgdaXiabbkdaYiabbccaGiabb2gaTjabb+gaVjabb6gaUjabbsha0jabbIgaOjabbohaZjabbccaGiabb+gaVjabbAgaMjabbccaGiabbggaHjabbEgaNjabbwgaLjabbccaGiabbEha3jabbIgaOjabb+gaVjabbccaGiabbkhaYjabbwgaLjabbogaJjabbwgaLjabbMgaPjabbAha2jabbwgaLjabbsgaKjabbccaGiabbkcaIiabbIfayjabbkcaIiabbccaGiabbohaZjabbogaJjabbIgaOjabbwgaLjabbsgaKjabbwha1jabbYgaSjabbwgaLjabbsgaKjabbccaGiabbAha2jabbggaHjabbogaJjabbogaJjabbMgaPjabb6gaUjabbwgaLbqaaiabbweafjabbohaZjabbsha0jabbMgaPjabb2gaTjabbggaHjabbsha0jabbwgaLjabbsgaKjabbccaGiabb6gaUjabbwha1jabb2gaTjabbkgaIjabbwgaLjabbkhaYjabbccaGiabb+gaVjabbAgaMjabbccaGiabbogaJjabbIgaOjabbMgaPjabbYgaSjabbsgaKjabbkhaYjabbwgaLjabb6gaUjabbccaGiabbwha1jabb6gaUjabbsgaKjabbwgaLjabbkhaYjabbccaGiabbgdaXiabbkdaYiabbccaGiabb2gaTjabb+gaVjabb6gaUjabbsha0jabbIgaOjabbohaZjabbccaGiabb+gaVjabbAgaMjabbccaGiabbggaHjabbEgaNjabbwgaLbaacqGHxdaTcqaIXaqmcqaIWaamcqaIWaamaaa@DAE3@

The denominators (estimated numbers of children under 12 months of age in 2000 and 2003) were calculated from the 2002 population census data [18] following the assumption that 4% of the entire population is under 12 months of age and the general level of population growth is 2.5% [19].

District infant immunisation coverage rates for the four vaccines (BCG, DPT3, POV3 and measles) were calculated using the reported number of children under 12 months of age who received scheduled vaccines by district and the estimated number of children under 12 months of age by district. The variables were calculated individually for the years 2000 and 2003.

A focus group discussion was held with eight Afghan experts in health, politics and media to identify factors exacerbating the poor security in Afghanistan during the years of the study from 2000 to 2003. The experts discussed the components defining the lack of security in Afghanistan from 2000–2003, and the level of insecurity in each of the seven regions of the country, rated as highly insecure (+++), insecure (++) or occasionally insecure (+).

The following EPI resources-related variables were retrieved from the national EPI database: numbers of male and female vaccinators per 100,000 infants, number of fixed EPI centres per 100,000 infants, and percentage of vaccinators paid less than 80 US dollars per month among all vaccinators in each district.

### Analysis

Spearman correlation analysis was performed to compare immunisation coverage data for different vaccines individually in 2000 and 2003 to determine the consistency of the obtained coverage data.

We used four categories of immunisation coverage percentage for further analysis: (A) less than 1%, (B) 1% or greater and less than 50%, (C) 50% or greater and less than 80% and (D) 80% or greater. Districts with less than 1% coverage were due to lack of reporting or lack of services in those districts. A coverage rate of 80% was used for this categorization in accordance with the WHO recommendation [20]. The numbers of districts in each of the four categories in 2000 and 2003 were compared by chi-square test.

Progress toward satisfying 80% coverage from 2000 to 2003 in each of the 7 regions for each of the four scheduled immunisations was examined. Changes in categories from 2000 to 2003 in the 7 regions were examined for statistical significance by chi-square test; percentage satisfaction of 80% coverage in 2000 and 2003 was tested by chi-square test in individual regions for each immunisation.

A multivariate logistic regression model was applied to examine the independent association between insecurity and immunisation coverage after adjustment for the influence of EPI resources.

The geographic distributions of district immunisation coverage of BCG, DPT3, OPV3 and measles vaccine were presented using Arch GIS (ESRI, Inc., Redlands, CA) version 8.3 and WHO Health Mapper Data Manager Software version 3, which were already adopted for information and mapping of public health in Afghanistan. The district boundaries were defined on the Arch GIS database for all districts for the present analysis.

## Results

The results of the present study indicated that among the 331 districts of Afghanistan, the number of districts reporting immunisation coverage increased from 223 (67%) in 2000 to 297 (90%) in 2003. The Spearman correlation coefficients between immunisation coverage in both years were ≥ 0.88 for the four different immunisations, BCG, DPT3, OPV3 and measles.

During the four-year period of the study, the national coverage rates for BCG, DPT3, OPV3 and measles vaccines increased from 50.9 to 75.2%, 34.5 to 59.9%, 35.5 to 59.9% and 39.5 to 55.3%, respectively. Progress was greater for DPT3 than BCG or measles vaccinations, and measles coverage showed the lowest progress. The number of EPI fixed centres increased from 429 in 2000 to 722 in 2003 and the number of vaccinators also increased from 860 to 1400 in the same period.

Table 1 shows the number of districts by coverage percentage categories for BCG, DPT3, OPV3 and measles for infants in 2000 and 2003. The percentages of districts with less than 1% coverage for BCG, DPT3, OPV3 and measles decreased significantly from 2000 to 2003 (P < 0.001, P < 0.001, P = 0.039, P < 0.001). In each of the year 2000 and 2003, percentages of districts with less than 1% coverage were similar across four different immunisations (P > 0.05). The percentages of districts showing coverage of 50% or greater for BCG, DPT3, OPV3 and measles vaccinations were 44.7%, 25.4%, 28.4% and 32.6% in 2000 and 68.0%, 56.4%, 56.7% and 50.6% in 2003, respectively. Progress from 2000 to 2003 was statistically significant for all four immunisations (P < 0.05).

**Table 1:** Number of districts by coverage percentage categories for BCG, DPT3, OPV3 and measles for infants in 2000 and 2003, Afghanistan.

**Immunisation**	**Coverage % categories**	**2000**		**2003**		
		Number of districts (%)		Number of districts (%)		*P *value
**BCG**	<1	107	(32)	36	(11)	<0.001
	1≤ to <50	76	(23)	70	(21)	
	50≤ to <80	51	(15)	70	(21)	
	80≤	97	(29)	154	(47)	
**DPT3**	<1	113	(34)	34	(10)	<0.001
	1≤ to <50	134	(40)	110	(33)	
	50≤ to <80	33	(10)	90	(27)	
	80≤	51	(15)	96	(29)	
**OPV3**	<1	118	(35)	35	(10)	0.039
	1≤ to <50	119	(36)	108	(33)	
	50≤ to <80	32	(10)	92	(28)	
	80≤	62	(19)	95	(29)	
**Measles**	<1	106	(32)	33	(10)	<0.001
	1≤ to <50	111	(34)	131	(40)	
	50≤ to <80	42	(13)	85	(26)	
	80≤	66	(20)	82	(25)	

BCG: Bacillus Calmette-Guerin vaccine, DPT3: the third dose of Diphtheria-Pertusis-Tetanus vaccine, OPV3: the third dose of Oral Polio vaccine

The total number of subject districts was 331. Some of the subtotals of number of districts may not be equal to 331 due to un-reporting from some districts.

*P *value is for the difference in percentage of districts less than 1% coverage in 2000 and 2003.

**Figure 1:**
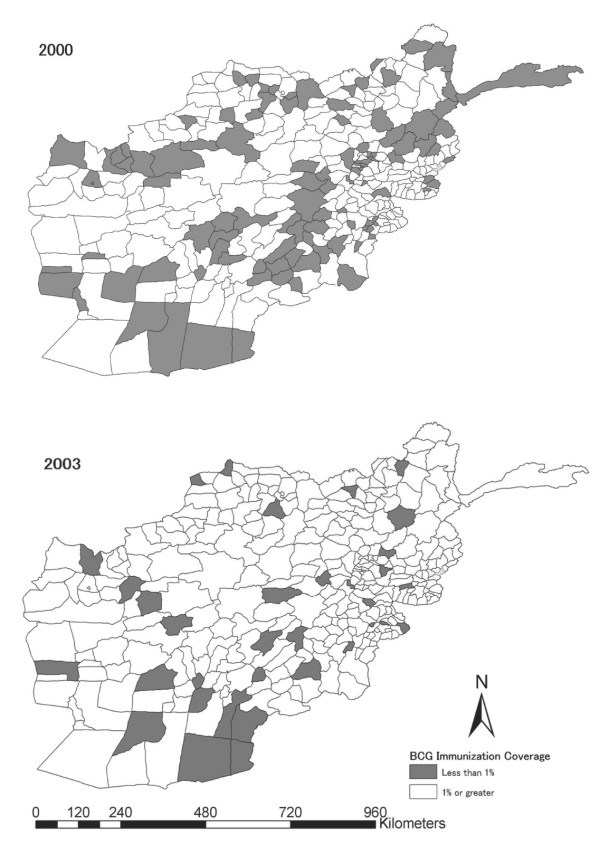
Geographic distribution of districts of non-reporting on BCG immunisation to infants, Afghanistan, 2000–2003.

As shown in Figure 1, there was an overall reduction in number of districts that did not report BCG immunisation from 2000 to 2003. Some districts reported BCG immunisation in 2000 but failed to do so in 2003.

Table 2 shows the number of districts divided according to the category of changes in coverage percentage stratified by 80% from 2000 to 2003 by the 7 geographic regions. For BCG immunisation, 18% to 31% of the districts in individual regions showed progress from coverage of less than 80% to 80% or greater. Statistically significant progress in the percentage of districts satisfying 80% coverage of BCG vaccination was observed for Central, Eastern, Northern and Southeast regions. For DPT3, OPV3 and measles immunisation, 4% to 37%, 6% to 46% and 4% to 23% of the districts in individual regions made progress from coverage of less than 80% to 80% or greater. The distribution of four categories according to changes in percentage coverage stratified by 80% was significantly different across the 7 regions studied for all four selected immunisations.

**Table 2:** Number of districts by categories of changes in coverage percentage, stratified by 80%, for BCG, DPT3, OPV3 and measles for infants from 2000 to 2003, by geographic regions in Afghanistan.

**Immunisation**	Categories of changes in immunisation coverage % 2000 to 2003	**National **N (%)		**Central **N (%)		**Eastern **N (%)		**Northeast **N (%)		**Northern **N (%)		**Southeast **N (%)		**Southern **N (%)		**Western **N (%)	
									
**BCG**	≥ 80 to ≥ 80	71	(22)	8	(17)	24	(56)	5	(11)	7	(16)	15	(25)	4	(8)	8	(19)
	< 80 to ≥ 80	82	(25)	13	(28)	10	(23)	10	(23)	8	(18)	18	(31)	10	(20)	13	(31)
	≥ 80 to < 80	24	(7)	0	(0)	1	(2)	6	(14)	4	(9)	4	(7)	4	(8)	5	(12)
	< 80 to < 80	151	(46)	25	(54)	8	(19)	23	(52)	26	(58)	22	(37)	31	(63)	16	(38)
*Difference in changes across 7 regions*			###														
*Difference in 80% coverage in 2000/2003 *respective regions			**		**		**				*		*				
**DPT3**	≥ 80 to ≥ 80	28	(8)	2	(4)	16	(39)	0	(0)	1	(2)	6	(10)	1	(2)	2	(5)
	< 80 to ≥ 80	68	(21)	8	(17)	15	(37)	6	(14)	10	(22)	18	(30)	2	(4)	9	(21)
	≥ 80 to < 80	22	(7)	0	(0)	1	(2)	3	(7)	3	(7)	7	(12)	3	(6)	5	(12)
	< 80 to < 80	211	(64)	36	(78)	9	(22)	35	(80)	32	(70)	29	(48)	44	(88)	26	(62)
*Difference in distribution across 7 regions*			###														
*Difference in 80% coverage in 2000/2003 respective regions*			**		*		*										
**OPV3**	≥ 80 to ≥ 80	24	(8)	2	(4)	12	(29)	1	(2)	1	(2)	8	(13)	0	(0)	0	(1)
	< 80 to ≥ 80	70	(21)	7	(15)	19	(46)	6	(14)	11	(24)	16	(27)	3	(6)	8	(19)
	≥ 80 to < 80	37	(11)	3	(7)	1	(2)	5	(11)	7	(15)	9	(15)	7	(14)	5	(12)
	< 80 to < 80	197	(60)	34	(74)	9	(22)	32	(73)	27	(59)	27	(45)	40	(80)	28	(67)
*Difference in distribution across 7 regions*			###														
*Difference in 80% coverage in 2000/2003 respective regions*			*				*										
**Measles**	≥ 80 to ≥ 80	36	(11)	4	(9)	16	(37)	2	(5)	3	(6)	8	(13)	1	(2)	2	(5)
	< 80 to ≥ 80	46	(14)	5	(11)	8	(19)	4	(9)	5	(11)	14	(23)	2	(4)	8	(19)
	≥ 80 to < 80	29	(9)	1	(2)	4	(9)	3	(7)	3	(6)	81	(13)	2	(4)	8	(19)
	< 80 to < 80	217	(66)	35	(78)	15	(35)	34	(79)	35	(76)	30	(50)	44	(90)	24	(57)
*Difference in distribution across 7 regions*			###														
*Difference in 80% coverage in 2000/2003 respective regions*			**		**		**				*						

### *P *< 0.001, distribution of 4 categories of changes in coverage % in 7 regions; * *P *< 0.05, ** *P *< 0.01 distribution of districts satisfied 80% coverage in 2000 and 2003

BCG: Bacillus Calmette-Guerin vaccine, DPT3: the third dose of Diphtheria-Pertusis-Tetanus vaccine, OPV3: the third dose of Oral Polio vaccine. The total number of subject districts was 331. Some of the subtotals of number of districts may not be equal to 331 due to un-reporting from some districts.

**Figure 2:**
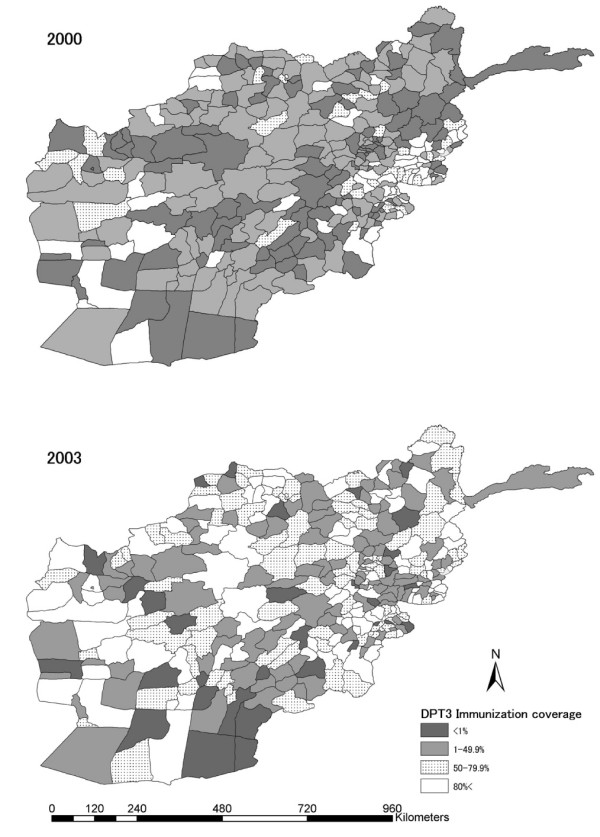
Geographic distribution of districts by categories of infant immunisation coverage for DPT3, Afghanistan, 2000–2003.

Figure 2 shows the geographic distribution of districts divided according to categories of infant immunisation coverage for DPT3. As shown in the figure, overall progress was observed in infant immunisation coverage by category. Similar degrees of progress in geographic distribution of districts by category of infant immunisation coverage for OPV3 and measles vaccination were observed.

Table 3 shows the level of insecurity and EPI resources by regions. One region was rated as highly insecure, 2 regions were rated as insecure, and 4 regions were rated as occasionally insecure in the period from 2000–2003.

**Table 3:** Level of insecurity and resources for immunisations by geographic regions in Afghanistan.

Regions	Central	Eastern	Northeast	Northern	Southeast	Southern	Western
* Insecurity	+	+	++	++	+	+++	+
							
§Resources							
Male and female vaccinators per 100,000 infants	198	231	157	171	177	261	146
Male vaccinators per 100,000 infants	129	184	135	132	153	215	120
Female vaccinators per 100,000 infants	69	47	22	39	24	47	26
EPI fixed centres per 100,000 infants	89	119	79	86	93	126	72
% of vaccinators with salary in US$ < 80	27	65	18	37	40	57	33

* Insecurity is classified as highly insecure (+++), insecure (++) and occasionally insecure (+) during years 2000 to 2003.

§ Number of Expanded Program on Immunisation resource by 100,000 infants in each region

Table 4 shows the associations between level of insecurity and infant immunisation. A rating of relatively secure was significantly associated with satisfaction of 80% coverage, regardless of available resources for EPI in the regions. None of the resource variables were associated with coverage for any of the four selected immunisations.

**Table 4:** Association between level of insecurity and infant immunisation coverage 80% and more by adjustment of immunisation resources in Afghanistan, 2003.

**Insecurity levels**	**BCG**			**DPT3**			**OPV3**			**Measles**		
				
	OR	(95% CI)	***P***	OR	(95% CI)	***P***	OR	(95% CI)	***P***	OR	(95% CI)	***P***
Highly insecure (+++)	(ref)			(ref)			(ref)			(ref)		
Insecure (++)	1.26	(0.57, 2.77)	0.57	4.17	(1.06, 16.38)	**0.04**	3.09	(1.03, 9.29)	**0.04**	4.02	(0.95, 16.99)	0.06
Occasionally insecure (+)	3.13	(1.56, 6.31)	**0.00**	12.16	(3.40, 43.55)	**0.00**	2.99	(1.21, 7.42)	**0.02**	10.31	(2.71, 39.23)	**0.00**

*Adjusted by number of total vaccinators, number of female vaccinators, and number of EPI fixed centers per 100,000 infants and percentage of vaccinators with salary less than US$ 80 per month.

## Discussion

**Figure 3:**
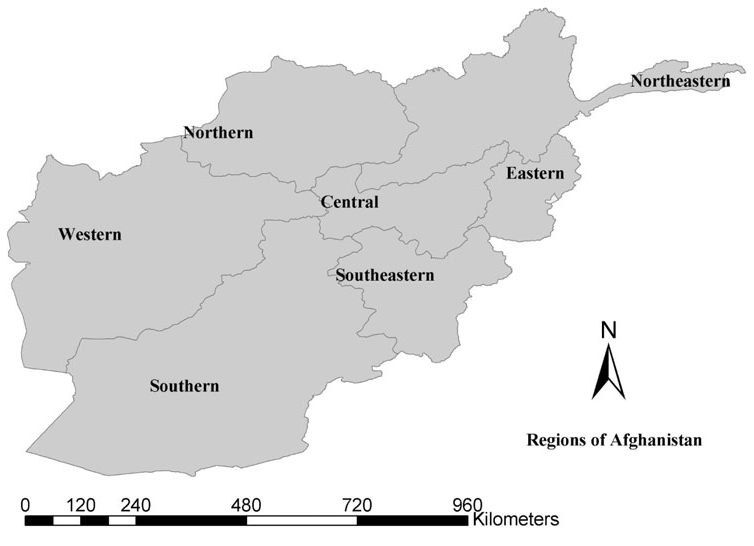
Seven regions in Afghanistan.

The number of districts in Afghanistan reporting immunisation coverage increased substantially during the four-year period from 2000–2003. Progress in BCG immunisation coverage was observed in all 7 regions examined in the present study (see figure 3), although unequal distribution to satisfy 80% coverage remained. Progress in DPT3, OPV3 and measles immunisation differed among regions, in addition to the unequal distribution of respective immunisation coverage in 2000. The lack of security played a significant role in the low coverage of immunisation regardless of the availability of immunisation services.

The high degree of progress in infant immunisation coverage in Afghanistan could be explained partly by increased donor support, which intensified from 2001, as well as to the country's political commitment to the immunisation programs [21]. These conditions are generally regarded as having facilitated smooth implementation of the programs by national, regional and local EPI partners. To further advance immunisation services, the year 2005 was declared the "Year of Child Immunisation" by the Ministry of Public Health, Afghanistan [22]. The results of the present study indicated that although Afghanistan achieved significant progress in immunisation coverage during the four years from 2000 to 2003, its overall coverage rates still do not satisfy the international standard of 80% coverage as defined by the WHO.

The coverages of DPT3 and OPV3 were very similar to each other at both time points in the present study. This was due to the immunisation schedule in Afghanistan, which recommended administration of both vaccines, *i.e*., DPT1 and OPV1, at a single contact with the child. Continuation of this strategy seems to work well in countries where resources are scarce and frequent contact with the child is difficult.

Afghanistan conducted nationwide measles catch-up and follow-up campaigns in 2002 and 2003, respectively: these campaigns were successful in achieving vaccination of approximately 96% of the children in the target populations [23]. According to the national guidelines, measles vaccination was administrated in a single dose. This single-dose policy was suggested to be responsible for the lowest degree of progress for measles vaccination observed in the present study, because in this policy children that had received the measles vaccine in the campaign were not eligible for the routine immunisation schedule.

One of the major strength of the present study was that we analysed the progress in infant immunisation coverage over time for all districts and regions of the country. The strong correlations of immunisation coverage by district among different immunisation schedules were regarded as consistent and reliable coverage data for each district. In addition, we also visualised the coverage distribution at the district level; our results indicated a wide degree of inequality between different parts of the country despite overall good progress. Finally, health service information was integrated into the GIS database. The further potential of the use of GIS with health information was recognised to strengthen the country's mapping of the population at risk, targeting focal areas for interventions and planning of the national health system.

Countries and regions facing conflict are characterised by violence, poverty, destruction of health infrastructure and large-scale population displacement [17]. Afghanistan has experienced such conflict since the late 1970s and is now considered to be experiencing post-conflict conditions. These conditions have had a marked impact on the delivery of health service programs in Afghanistan.

The results of the present study indicated that security within a country is an important factor for delivering immunisation services. A report indicates that the southern region of the country is under the poorest conditions with regard to security [24]. Despite the presence of relatively large number of vaccinators and EPI centres per population, immunisation coverage in this region failed to increase from 2000 to 2003. The influence of political instability on child health in general has been noted [25]. The independent significant impact of lack of security on child immunisation indicates the difficulties in public health service efforts aimed at reaching all potential beneficiaries under conditions of armed conflict.

Traditional culture plays an important role in participation in vaccination programs for children, and can act as a barrier preventing mothers making contact with male vaccinators; in the majority of cases in Afghanistan, it is necessary for mothers to have permission from the head of the family, and sometimes they are not allowed to go to the health centre without a male escort. A study conducted in the neighbouring country of Pakistan indicated that a lack of female autonomy, especially in rural areas in which they are not allowed to visit health facilities alone or even to make decisions regarding the spending of money on healthcare, limits their access to healthcare [26].

Based on our observations in the present study, we suggest that the introduction of a simple poorly designed solution will not overcome the problem of low immunisation coverage in insecure areas. The problem is multidimensional, and it is necessary to bring together the program policy makers, local mangers, and key people within the community to find a practical solution to increase the coverage even under conditions of insecurity. It may be possible to employ trained indigenous health workers from the same district who will be able to work under insecure conditions and will be better informed regarding the local situation. Moreover, avoiding delays in payment of monthly salaries and providing extra incentives to workers may further motivate these employees to work hard. Further research is necessary to determine the underlying causes of low immunisation coverage in insecure regions, such as the effects of insecurity on the mobility of vaccinators, community awareness and socioeconomic status.

The results reported here suggested the widening of inequality in health developments among regions, despite substantial improvements in immunisation coverage in national average figures in Afghanistan. Our findings clearly indicated the negative impact of insecurity on infant immunisation despite resource allocation. Furthermore, the results of this study demonstrated the importance of geographic analysis of health practice data in countries with limited resources under insecure conditions.

## Conclusion

Although progress was observed in all 7 regions in Afghanistan examined in the present study, geographic inequalities in the improvements remain a cause for concern. Our results indicated that security within a country is an important factor in the delivery of immunisation services. There results will be of use in aiding policymakers identify areas that require further attention in immunisation programs.

## Competing interests

The author(s) declare that they have no competing interests.

## Authors' contributions

TM designed the study, analysed the data and drafted the manuscript. KN participated in data analysis, structure and edited the manuscript. MK and KS took part in database preparation and revison of the drafts. TT supervised the data analysis and writing. All authours read and approved the final manuscript.
